# Usefulness of combined screening methods for rapid detection of falsified and/or substandard medicines in the absence of a confirmatory method

**DOI:** 10.1186/s12936-019-3045-y

**Published:** 2019-12-05

**Authors:** Kwabena Frimpong-Manso Opuni, Henry Nettey, Marvin Adjei Larbi, Salome Naa Amerley Amartey, Gifty Nti, Abraham Dzidonu, Patrick Owusu-Danso, Nicholas Amoah Owusu, Alexander Kwadwo Nyarko

**Affiliations:** 1 Department of Pharmaceutical Chemistry, School of Pharmacy, University of Ghana, Accra, Ghana; 2 Department of Pharmaceutics and Microbiology, School of Pharmacy, University of Ghana, Accra, Ghana; 3 Apotheke Doc Morris, Avantisalle 152, 6422 RA Heerlen, The Netherlands; 4 Laboratory Services Department, Food and Drugs Authority, Accra, Ghana; 5 Department of Pharmacology and Toxicology, School of Pharmacy, University of Ghana, Accra, Ghana

**Keywords:** Anti-malarial, Falsified medicines, Substandard medicines, Screening and confirmatory methods

## Abstract

**Background:**

The influx of substandard and falsified medicines is a global public health challenge and its rapid detection is a key solution to the menace. This study used three screening methods and one confirmatory method for the quality assessment of 25 batches of artemether/lumefantrine dosage forms from the Ghanaian market to test that combined screening methods only can rapidly detect substandard and/or falsified medicines in areas where confirmatory methods may not be available.

**Methods:**

The quality of artemether/lumefantrine tablet products obtained from pharmacies and licensed chemical seller shops within the Accra metropolis in Ghana were analysed using three screening methods (GPHF Minilab, Colorimetry and Counterfeit Drug Indicator) and one confirmatory method (high-performance liquid chromatography).

**Results:**

The results showed that 18/25 batches of the artemether/lumefantrine samples passed using the combined screening and confirmatory methods and 5/25 batches of the artemether/lumefantrine samples failed using the combined screening and confirmatory methods. However, 1/25 batch of the artemether/lumefantrine samples failed using the combined screening methods but passed using the confirmatory method. Also, 1/25 batch of the artemether/lumefantrine samples passed using the combined screening methods but failed using the confirmatory method. This notwithstanding, the combined screening methods and the confirmatory method provided equivalent quality assessment profiles for 23/25 (92%) batches of the artemether/lumefantrine tablet products. Out of the 6 samples that failed the confirmatory test, 1/6, 2/6, and 3/6 failed on the high (> 110%), low (< 90%), and no active ingredient (0%), respectively. The sensitivity of Minilab, colorimetric, CoDI, and the combined screening methods at 95% confidence level were 0.5 ± 0.57, 0.83 ± 0.33, 0.75 ± 0.49, and 0.83 ± 0.33, respectively. Also, the specificity of Minilab, colorimetric, CoDI, and the combined screening methods at 95% confidence level were 1.00, 0.95 ± 0.10, 1.00, and 0.95 ± 0.10, respectively.

**Conclusion:**

The combined screening methods may be used for rapid detection of falsified and/or substandard medicines without using a confirmatory method. However, additional research on the best combinations of screening devices/methods to rapidly detect the quality of medicines is recommended.

## Background

Substandard and falsified medicines may be harmful to patients. They cause treatment failure to diseases, and reduce confidence in medicines, healthcare providers and health systems [1, 2]. The presence of substandard and falsified medicines is a global challenge and developing countries bear the highest risk. It has been estimated that approximately 10% of medical products in low- and middle-income countries are substandard or falsified [3]. Although, a wide range of medicines (generics and innovator) including vaccines and diagnostics have been reported as substandard and falsified, anti-malarials and antibiotics are the most frequently reported [4]. For example, the prevalence of falsified and substandard anti-malarial medicines has been between 20 and 64% [5, 6]. Also, various studies have shown the influx of falsified and substandard artemether/lumefantrine formulations on the African and Ghanaian markets [2, 7–10]. Various reports suggest that falsified and substandard medicines have contributed to a significant increase in antimicrobial resistance [8, 11, 12]. There is, therefore, an urgent need to perform quality assessment of medicines on the market for rapid detection of medicines which are falsified and/or substandard. This will reduce and/or prevent the circulation of falsified and substandard medicines in developing countries and reduce the public health challenges associated with the menace.

The quality of medicines is normally assessed by using either screening [13, 14] and/or confirmatory [13] methods. There are over 40 reported screening methods for quality assessment of medicines [13, 15] such as Global Pharma Health Fund (GPHF) Minilab kit [16, 17], Counterfeit Drug Indicator (CoDI) [18], colorimetry [19], CD3+ [20], TruScan handheld Raman spectrometer [16], and reflectance infrared spectroscopy [21]. Although, some screening devices such as Minilab, colorimetry, and CoDI are inexpensive, others, which are mostly spectrophotometers [TruScan, Progeny, MicroPhazir, and Near-Infrared (NIR)] are very expensive [15]. Also, some screening devices, e.g., the CoDI, and the paper analytical devices (PADs) are simple and non-sophisticated and can easily be applied in the field, but the simplicity of other devices depends on what you want to do with them, especially for the spectrophotometers. For example, Minilab requires some laboratory experience and continuous training [15]. Screening devices/methods sometimes have issues of sensitivity and specificity i.e. false positive results [22].

Several confirmatory methods are available for quality assessment of medicines, among which high performance liquid chromatography is the gold standard [13]. However, the confirmatory methods are expensive, sophisticated, require extensive training and well-equipped laboratory for effective application [22]. Although, there is no single screening method capable for detecting falsified and substandard medicines, there is a high chance of detecting falsified and substandard medicines using combined screening methods (i.e. two or more screening methods) since each screening method is based on unique set of principle(s).

The objective of this work is to test that combining the results of screening methods will identify falsified and/or substandard drugs. The approach of this study utilized a sample of five batches of artemether/lumefantrine tablets of known quality status and perform quality analysis using three screening methods (GPHF Minilab, Colorimetry, and Counterfeit Drug Indicator) and one confirmatory method [high performance liquid chromatography) (HPLC)]. Thereafter, a sample of twenty batches of artemether/lumefantrine tablets of unknown quality status were analysed using the same approach. This approach was chosen since the 5 samples were obtained from Centers for Disease Control and Prevention (CDC) with known quality status, which were used to check the suitability of the HPLC method before applying it to the other 20 samples obtained from pharmacies and licensed chemical shops.

This strategy, which uses different screening methods can potentially be useful in jurisdictions where gold standard methods such as HPLC, which are expensive, laborious, and requires specialized skills are not readily available. Additionally, since falsified and/or substandard drugs is a public health menace, this approach will be able to rapidly detect these falsified and/or substandard anti-malarial drug products in the market and improving the quality of life.

## Methods

### Materials

Global Pharma Health Fund (GPHF) Minilab Kit (Merck, Germany), Artemether reference standard (USP grade, Lot H0M313, USP, Rockville, USA), Lumefantrine reference standard (USP grade, Lot G0L394, USP, Rockville, USA), Acetonitrile (ACN) (HPLC grade, PROLABO^®^ VWR International, France), Trifluoroacetic acid (TFA) (Batch 10812LH, Sigma-Aldrich Inc., USA), 1-hexane sulfonic acid sodium salt hydrate (Lot A0370098, ACROS organics, UK), Glacial acetic acid, Ethyl acetate, 85% Phosphoric acid, Methanol, Acetic acid, Congo red, Counterfeit Detection Indicator (CDC, Atlanta, GA).

### Sampling of artemether/lumefantrine tablet products

Seven artemether/lumefantrine tablet products registered by the Food and Drugs Authority-Ghana were obtained from pharmacies and licensed chemical seller shops within the Accra metropolis, Ghana. All the seven artemether/lumefantrine tablet products comprised three batches each, except one product which was two batches. Additionally, five artemether/lumefantrine tablet products were obtained from CDC which had previously been confirmed to be either of good quality, falsified or substandard (Table 1). The medicines were stored at the recommended manufacturer’s storage conditions.

**Table 1:** Profile of drug samples

Brand name	Manufacturer	Drug code	Batch	Man. date	Expiry date	Label claim
Artefan	Ajanta, India	AT	1	Jun-17	May-19	Artemether 20 mg/lumefantrine 120 mg
			2	Jun-17	May-19	Artemether 20 mg/lumefantrine 120 mg
			3	Jun-17	May-19	Artemether 20 mg/lumefantrine 120 mg
Coartem Green Leaf	Novartis, Switzerland	CG	1	Sep-17	Aug-19	Artemether 20 mg/lumefantrine 120 mg
			2	Apr-17	Mar-19	Artemether 20 mg/lumefantrine 120 mg
			3	Sep-17	Aug-19	Artemether 20 mg/lumefantrine 120 mg
Coartem Dispersible	Novartis, Switzerland	CD	1	Apr-17	Mar-19	Artemether 20 mg/lumefantrine 120 mg
			2	Dec-16	Nov-18	Artemether 20 mg/lumefantrine 120 mg
			3	May-17	Apr-19	Artemether 20 mg/lumefantrine 120 mg
Combiart	Strides Shasun, India	CO	1	May-16	Apr-18	Artemether 20 mg/lumefantrine 120 mg
			2	May-17	Apr-19	Artemether 20 mg/lumefantrine 120 mg
			3	May-17	Apr-19	Artemether 20 mg/lumefantrine 120 mg
Lonart Forte	Bliss GVS, India	LO	1	Feb-17	Jan-19	Artemether 40 mg/lumefantrine 240 mg
			2	Feb-17	Jan-19	Artemether 40 mg/lumefantrine 240 mg
			3	Sep-16	Aug-18	Artemether 40 mg/lumefantrine 240 mg
Danamether	Danadams, Ghana	DA	1	Sep-17	Sep-19	Artemether 20 mg/lumefantrine 120 mg
			2	Nov-17	Nov-19	Artemether 20 mg/lumefantrine 120 mg
			3	Sep-17	Sep-19	Artemether 20 mg/lumefantrine 120 mg
Gen-M	Genix pharma, Pakistan	GM	1	Nov-16	Nov-18	Artemether 20 mg/lumefantrine 120 mg
			2	May-17	May-19	Artemether 20 mg/lumefantrine 120 mg
Coartem	Novartis, USA	CV^a^	1	Jun-14	May-17	Artemether 20 mg/lumefantrine 120 mg
Coartem	Novartis, USA	CW^a^	1	Jan-13	Nov-15	Artemether 20 mg/lumefantrine 120 mg
Coartem	Novartis Saglik, Turkey	CX^a^	1	Aug-15	Jul-17	Artemether 20 mg/lumefantrine 120 mg
Coartem	Novartis, USA	CY^a^	1	Jan-12	Jan-16	Artemether 20 mg/lumefantrine 120 mg
Coartem	Novartis Saglik, Turkey	CZ^a^	1	Jun-15	May-17	Artemether 20 mg/lumefantrine 120 mg

^a^These samples were obtained from CDC and had previously confirmed to be of good quality, falsified or substandard

### Minilab analysis of artemether/lumefantrine tablet products

The analysis of the artemether/lumefantrine tablet products was performed using GPHF Minilab Kit as per manufacturer’s instructions [23, 24]. The GPHF Minilab kit contains the equipment, reference standards, and a manual of procedures to perform basic tests such as thin layer chromatography. The Minilab analysis includes physical examination of the tablets, disintegration test, and thin-layer chromatography, which are described in Additional file 1: Additional methods.

### Colorimetric analysis of artemether/lumefantrine tablet products

The artemether/lumefantrine tablet products were analysed using a colorimetric method as previously described [18] (Additional file 1: Additional methods, Additional file 2: Fig. S1). The International Pharmacopoeia limit of 90.0% to 110.0% for the assay of artemether/lumefantrine [25] was used to determine whether a sample passed or failed.

### CoDI analysis of artemether/lumefantrine tablet products

The artemether/lumefantrine tablets were analysed using a prototype Counterfeit Detection Indicator as previously described [18] (Additional file 1: Additional methods, Additional file 3: Fig. S2). The interpretation of the CoDI value was performed by setting the threshold for the CoDI value to 0.44–0.68. Any CoDI value outside the threshold was considered failed [18].

### HPLC analysis of artemether/lumefantrine tablet products

The analysis of the artemether/lumefantrine tablet products was performed using HPLC as previously described [25] (Additional file 1: Additional methods, Additional file 4: Table S1). The International Pharmacopoeia limit of 90.0% to 110.0% for the assay of artemether/lumefantrine was used to determine whether a sample passed or failed.

## Results

### The usefulness of combined screening methods on confirmed falsified artemether/lumefantrine tablets

In all, 25 artemether/lumefantrine tablet product batches were analysed using three screening methods and one confirmatory method. However, quality analysis of 5 batches of artemether/lumefantrine tablet products obtained from CDC was initially performed. The samples from CDC were used to check the suitability of the HPLC method applied for this study since the quality status of samples were already known. Thereafter, 20 samples obtained locally were also analysed using the same screening and confirmatory methods. Hence, the results from the 5 samples from CDC will first be presented, followed by that of the 20 samples.

**Fig. 1:**
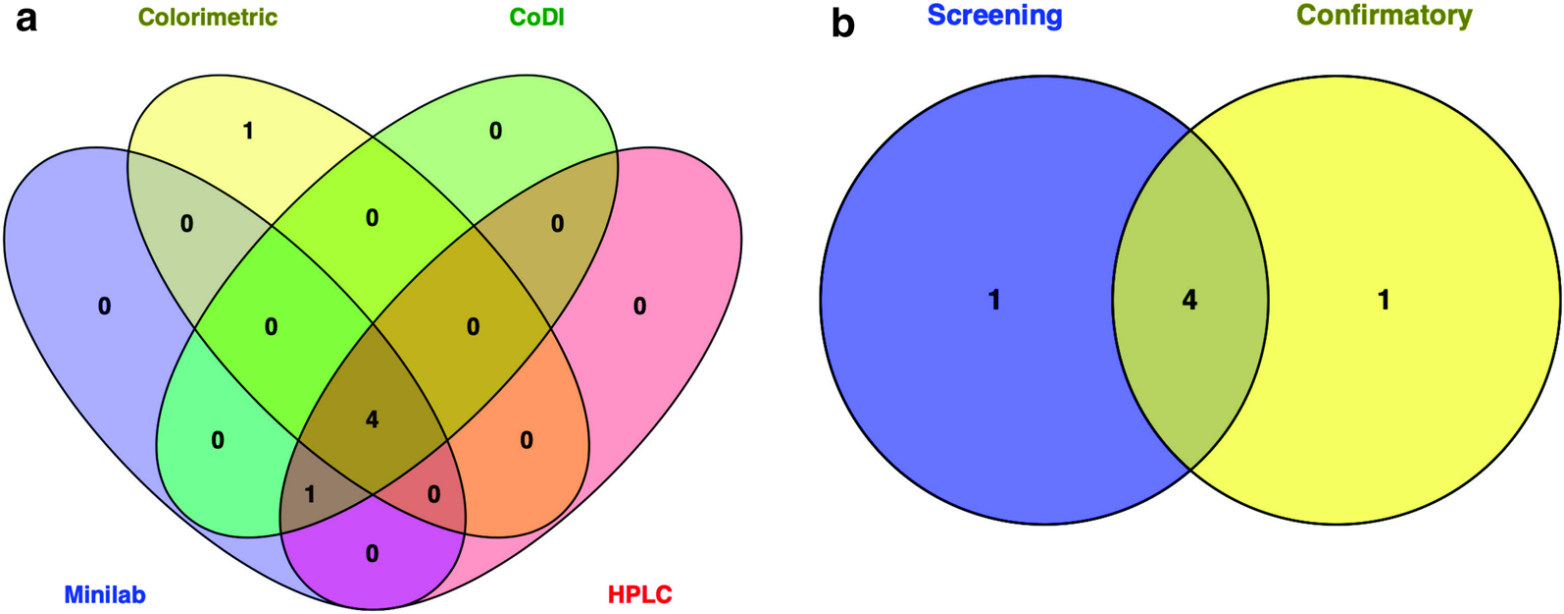
Summary of quality assessment of artemether/lumefantrine tablet products. **a** Venn diagram representation of Minilab, CoDI, colorimetric and HPLC methods. **b** Venn diagram representation of the combined screening methods compared to the confirmatory method. The numbers in the bubbles of the Venn diagram represents test results from the screening and confirmatory methods

Comparison of quality assessment results shows that 1/5 artemether/lumefantrine product passed using the combined screening methods, whilst 2/5 artemether/lumefantrine products also passed the confirmatory method. However, 1/5 artemether/lumefantrine product passed using both the combined screening methods and the confirmatory method (Table 2). Similarly, 4/5 artemether/lumefantrine products failed using the combined screening methods, whilst 3/5 artemether/lumefantrine products also failed the confirmatory method. However, 3 products of the artemether/lumefantrine tablets failed using both the combined screening methods and the confirmatory method (Table 2). Among the screening methods, 4/5 products showed similar results, whilst 5/5 products had two of the screening methods (CoDI and Minilab) agreeing (Fig. 1a). The results from the Minilab and CoDI experiments showed 100% agreement with that of the HPLC method. It was only one result from the colorimetric method which was different from that of the HPLC method (Fig. 1a). There were no defects of the all packaging materials and the tablets except the tablets of CY (Additional file 5: Table S2). The samples that failed using the confirmatory method also failed disintegration test of the Minilab (Additional file 5: Table S2).

**Table 2:** Results from four methods for artemether/lumefantrine tablets of known quality

Drug Code	Batch	Active ingredient	Minilab		Colorimetric		CoDI		HPLC	
			Error^b^ NMT 5%	Status (pass/fail)	Assay content (%)	Status (pass/fail)	CoDI Value	Status (pass/fail)	Assay Content (%)	Status (pass/fail)
CV^a^	1	Artemether	na	Failed	0.00	Failed	0.31	Failed	0.00	Failed
		Lumefantrine	na	Failed	0.00	Failed		Failed	0.00	Failed
CW^a^	1	Artemether	na	Failed	0.00	Failed	0.36	Failed	0.00	Failed
		Lumefantrine	na	Failed	0.04	Failed		Failed	0.00	Failed
CX^a^	1	Artemether	0	Passed	97.71	Passed	0.48	Passed	108.17 ± 1.67	Passed
		Lumefantrine	0	Passed	91.42	Passed		Passed	94.09 ± 0.10	Passed
CY^a^	1	Artemether	na	Failed	0.00	Failed	0.00	Failed	0.00	Failed
		Lumefantrine	na	Failed	0.00	Failed		Failed	0.00	Failed
CZ^a^	1	Artemether	0	Passed	96.83	Passed	0.50	Passed	102.85 ± 1.18	Passed
		Lumefantrine	0	Passed	84.94	Failed		Passed	90.58 ± 0.14	Passed

na: not applicable because sampling error could not be calculated due to no retardation factor (Rf) value for the test sample

^a^These samples were obtained from CDC and had previously confirmed to be of good quality, falsified or substandard

^b^Sampling error

As a whole, 4/5 (80%) of the artemether/lumefantrine products showed similar consensus between the combined screening methods and the confirmatory method (Fig. 1b). Of note, the results of the confirmatory method agree totally with the known data of the test samples. However, the results of the combined screening methods agree with 4 samples and in one case provided a false negative result (Fig. 1a). Upon completion of analysis of the 5 samples using the combined screening and confirmatory methods, the concept was applied to additional 20 artemether/lumefantrine products of unknown quality status.

### Quality assessment of artemether/lumefantrine tablets using the combined screening and confirmatory methods

The quality of 7 artemether/lumefantrine tablet products (comprising a total of 20 batches) was assessed using the same screening and confirmatory methods as indicated above. From the Minilab analysis, 20/20 of the artemether/lumefantrine tablet products passed the quality assessment including physical examination of the packaging materials and disintegration test (Additional file 5: Table S2). The colorimetry method showed that 18/20 of the artemether/lumefantrine tablet products passed the quality test, whilst 2/20 failed (Additional file 6: Table S3). The prototype CoDI device applied in this study was designed for the analysis of all uncoated tablets. However, it has a requirement that prior values for the original manufacturer (innovator) be established. This could not be done because we could not get the manufacturers (e.g. Novartis) to send us the samples we requested. As a result, the prototype CoDI device could not be used for the analysis of some of the artemether/lumefantrine products. Therefore, only 3 artemether/lumefantrine tablet product batches were analysed. The results indicated that 3/3 of the batches passed (Additional file 7: Table S4).

The artemether/lumefantrine products assayed using HPLC showed that 17/20 passed the quality assessment, whilst 3/20 failed (Additional file 8: Table S5).

The comparison of quality assessment presented in Table 3 shows that, 18/20 artemether/lumefantrine batches passed using the combined screening methods, whilst 17/20 artemether/lumefantrine batches also passed the confirmatory method.

**Table 3:** Test results for artemether/lumefantrine tablets of unknown quality using screening and HPLC methods

Drug code	Batch	Screening methods				HPLC
		Minilab	Colorimetric	CoDI	Combined	
AT	1	Passed	Passed	na	Passed	Passed
	2	Passed	Passed	na	Passed	Passed
	3	Passed	Passed	na	Passed	Passed
CG	1	Passed	Passed	Passed	Passed	Passed
	2	Passed	Failed	Passed	Failed	Failed
	3	Passed	Passed	Passed	Passed	Passed
CD	1	Passed	Passed	na	Passed	Passed
	2	Passed	Passed	na	Passed	Passed
	3	Passed	Passed	na	Passed	Passed
CO	1	Passed	Passed	na	Passed	Passed
	2	Passed	Passed	na	Passed	Passed
	3	Passed	Passed	na	Passed	Passed
LO	1	Passed	Passed	na	Passed	Passed
	2	Passed	Passed	na	Passed	Passed
	3	Passed	Passed	na	Passed	Failed
DA	1	Passed	Passed	na	Passed	Passed
	2	Passed	Passed	na	Passed	Passed
	3	Passed	Passed	na	Passed	Passed
GM	1	Passed	Failed	na	Failed	Failed
	2	Passed	Passed	na	Passed	Passed

na: not applicable

**Fig. 2:**
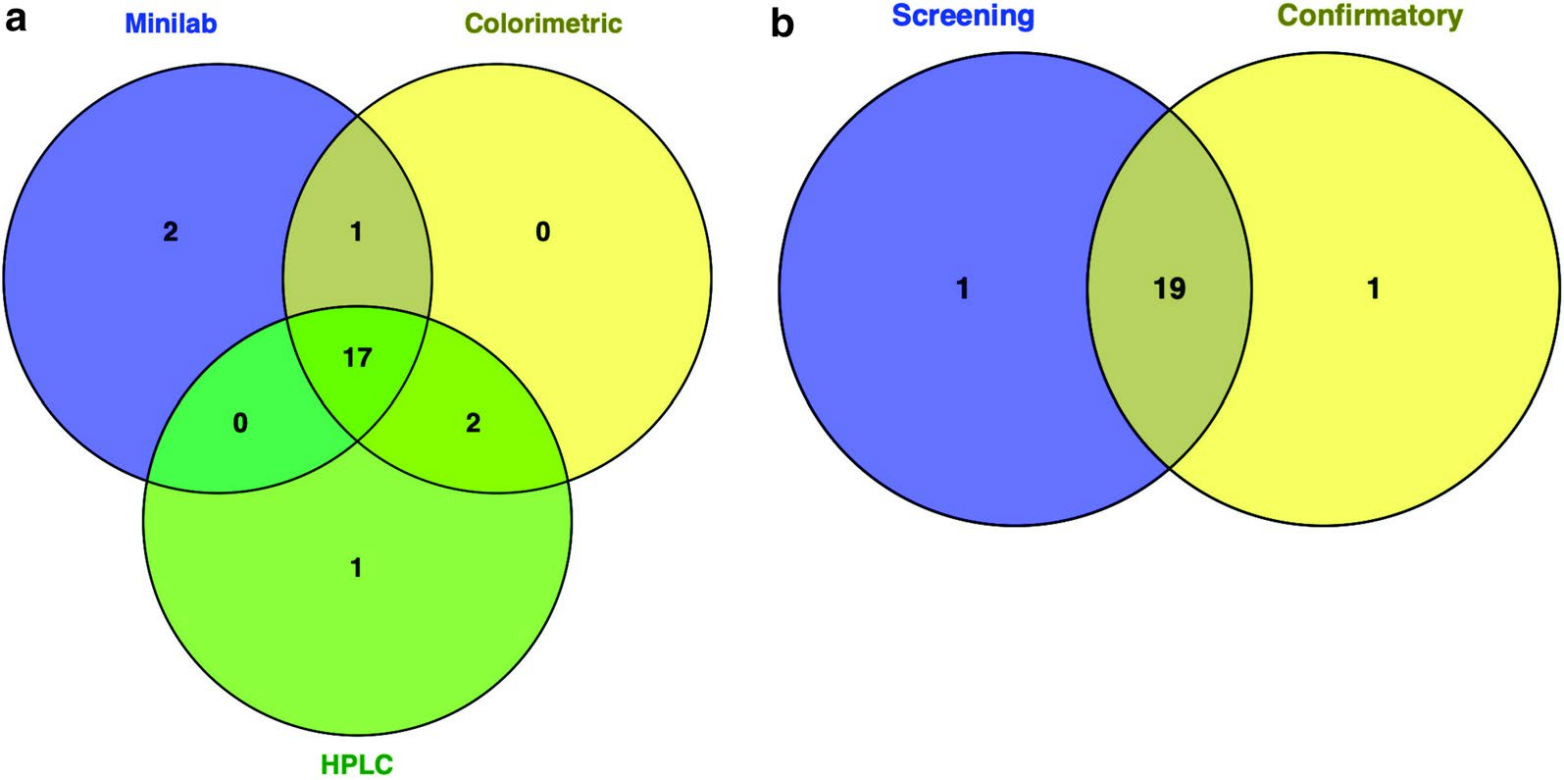
Summary of quality assessment of artemether/lumefantrine tablet products. **a** Venn diagram representation of Minilab, colorimetric and HPLC methods. **b** Venn diagram representation of the combined screening methods compared to the confirmatory method. The numbers in the bubbles of the Venn diagram represents test results from the screening and confirmatory methods

As a result, 17/20 batches of the artemether/lumefantrine products passed using both the combined screening methods and the confirmatory method. Similarly, 2/20 artemether/lumefantrine batches failed using the combined screening methods. Among the screening methods, 18/20 products showed similar results, whilst 2/20 products had varying results (Fig. 2a). As a whole, 19/20 (95%) of the artemether/lumefantrine batches showed strong consensus between the combined screening methods and the confirmatory method (Fig. 2b).

Pooling the results of all the samples together, 18/25 artemether/lumefantrine samples passed, and 5/25 artemether/lumefantrine samples failed using the combined screening and confirmatory methods. 1/25 artemether/lumefantrine samples failed using the combined screening methods but passed using the confirmatory method. Also, 1/25 artemether/lumefantrine samples passed using the combined screening methods but failed using the confirmatory method. Overall, the combined screening methods and the confirmatory method showed similar results (23/25, i.e., 92%) for the artemether/lumefantrine products.

### Characteristics and performance results of the screening methods

The main characteristics and performance results of the screening methods such as sensitivity and specificity were measured by comparing the results of screening methods to that of the HPLC method. Sensitivity is defined as the proportion of medicines that are determined as falsified/substandard by a screening device out of all the medicines detected as falsified/substandard by a reference method. On the other hand, specificity is defined as the proportion of medicines that are determined to be of good quality by a screening device out of all the medicines determined to be of good quality by a reference method [15, 20]. The false positive results of Minilab, colorimetric, CoDI, and the combined screening methods were 0, 1, 0, and 1, respectively (Table 4). Also, false negative results of Minilab, colorimetric, CoDI, and the combined screening methods were 3, 1, 1, and 1, respectively (Table 4). The sensitivity of Minilab, colorimetric, CoDI, and the combined screening methods at 95% confidence level were 0.50 ± 0.57, 0.83 ± 0.33, 0.75 ± 0.49, and 0.83 ± 0.33, respectively (Table 4). Additionally, the specificity of Minilab, colorimetric, CoDI, and the combined screening methods at 95% confidence level were 1.00, 0.95 ± 0.10, 1.00, and 0.95 ± 0.10, respectively (Table 4).

**Table 4:** Statistical performance of the screening methods

	Passed samples	Failed samples	False negative	True Positive	False positive	True negative	Sensitivity	95% CI	Specificity	95% CI
HPLC (n = 25)	19.00	6.00	–	–	–	–	–	–	–	–
Minilab (n = 25)	22.00	3.00	3.00	3.00	0.00	19.00	0.50	0.57	1.00	0.00
Colorimetric (n = 25)	19.00	6.00	1.00	5.00	1.00	18.00	0.83	0.33	0.95	0.10
CoDI (n = 5)	5.00	3.00	1.00	3.00	0.00	4.00	0.75	0.49	1.00	0.00
Combined screening methods (n = 25)	19.00	6.00	1.00	5.00	1.00	18.00	0.83	0.33	0.95	0.10

## Discussion

Many screening devices may be able to detect medicines with no or wrong active pharmaceutical ingredient (API). However, none have been shown to accurately quantify a diversity of APIs [13, 15]. This suggests that individual screening methods/devices will not be able to detect many falsified and/or substandard antimalarials. It is therefore necessary to identify the best combination of screening methods for the detection of substandard/falsified medicines. This study investigated the possibility of using three low cost (< $10,000.00) [13] screening methods (Minilab, CoDI, colorimetry) for detection of substandard/falsified anti-malarials.

The sensitivity of the Minilab screening method from this study at 95% confidence level (0.50 ± 0.57) is within the published values of 0.29 to 1.0 for either identification, dissolution, or content test. However, high sensitivity values or Minilab is normally reported for identification test [6, 20]. On the other hand, specificity of the Minilab screening method from this study at 95% confidence level (1.0) agrees with the reported value of 1.0 for identification and content test [20]. Although, the specificity of the Minilab method is high (1.0), its sensitivity (0.50 ± 0.57) makes it not amenable to be used alone for the detection of substandard/falsified medicines. Also, the Minilab method requires the use of consumables (solvents) that may expire and difficult to source in some countries [15]. Another challenge is that the Minilab method can only detect grossly substandard/falsified medicines as shown in our results and requires the expertise of at least a laboratory technician [13].

From a previous study using CoDI, 6 falsified and 12 good quality artemether/lumefantrine tablets were correctly discriminated [18], which suggests a high sensitivity and specificity. The specificity of CoDI from this work agrees with the published information, however, the sensitivity (0.75 ± 0.49) is below the reported literature. Since the prototype CoDI device applied in this study was designed for the analysis of uncoated tablets and also required samples from the innovator, only 6 samples were analysed. This limitation could affect the sensitivity of the method. Therefore, the CoDI device alone may not be able to detect substandard/falsified medicines although it requires minimal training prior to usage [13].

Colorimetric method has demonstrated high specificity (0.94–1.00) for all drugs [19], which agrees with the reported specificity from this study. The quantitative ability of the colorimetric method makes it amenable for detection of substandard/falsified medicines, since there are not many low-cost semi or quantitative screening methods available. The results of the colorimetric method were close to that of the reference method (HPLC) with the exception of one false positive and one false negative result. This shows that colorimetric screening method may be of great interest for public health through quality monitoring of substandard/falsified medicines. However, the colorimetric device requires the use of an oven and a trained laboratory technician [13].

Although, the goal of this study was to identify the best combination of screening methods for the detection of substandard/falsified medicines, the study results show that the colorimetry performed similarly as that of the combined screening methods. The 8% of samples wrongly detected by the screening methods/technologies is not acceptable for quality monitoring of medicines and has serious public health implications. Interestingly, the sample with tablet defects failed using all the screening and confirmatory methods, which suggests that screening methods that focus on packaging material and tablet inspection may compliment other screening methods for the detection of substandard/falsified medicines. Therefore, additional research work is needed to identify best combinations of existing screening devices/methods for the detection of substandard/falsified medicines. The limitation of this study was the small sample size coupled with only two APIs i.e., anti-malarial medicines (artemether/lumefantrine). More importantly, the findings from this work could inform policy on medicines regulation and guide Medicines Regulatory Authorities (MRAs) to choose the best screening methods/devices for their context, since none of the screening methods could independently performed compared to the reference method for the detection of substandard and falsified medicines.

## Conclusions

This work demonstrates the usefulness of combined screening methods for detection of falsified and/or substandard medicines in the absence of a confirmatory method. This notwithstanding, further work into possible best combinations of most appropriate screening devices/methods to detect the quality of medicines is needed.

## Supplementary information

**Additional file 1.** Additional methods.

**Additional file 2: Figure S1.** Colorimetric analysis of artemether/lumefantrine tablet products. IA: Samples in the 24-well plate before analysis with the colour software for artemether. IIA: Samples in the 24-well plate before analysis with the colour software for lumefantrine. IB: Samples in the 24-well plate during analysis with the MVHImage software for artemether. IIB: Samples in the 24-well plate during the analysis with the MVHImage software for lumefantrine.

**Additional file 3: Figure S2.** The prototype CoDI equipment. A: Different parts of the CoDI equipment. B: The CoDI equipment showing the slot and its operation.

**Additional file 4: Table S1.** Gradient elution programme.

**Additional file 5: Table S2.** Minilab test results for artemether/lumefantrine tablets of unknown quality

**Additional file 6: Table S3.** Assay of artemether/lumefantrine tablets of unknown quality using colorimetry.

**Additional file 7: Table S4.** Assay of artemether/lumefantrine tablets of unknown quality using Counterfeit Detection Indicator (CoDI).

**Additional file 8: Table S5.** Assay of artemether/lumefantrine tablets of unknown quality using HPLC.

## Data Availability

The datasets used and/or analysed during the current study are available from the corresponding author on reasonable request.

## Abbreviations

ACN: acetonitrile
CoDI: counterfeit drug indicator
GPHF: Global Pharma Health Fund
HPLC: high performance liquid chromatography
TFA: trifluoroacetic acid
USP: United States Pharmacopoeia
