# Supplementary material for: Usefulness of combined screening methods for rapid detection of falsified and/or substandard medicines in the absence of a confirmatory method
Source: Malar J. 2019 Dec 5;18:403. doi: 10.1186/s12936-019-3045-y (PMC6896689; doi:10.1186/s12936-019-3045-y)
Supplement: Supplementary file 1 — Additional file 1. Additional methods. [file 12936_2019_3045_MOESM1_ESM.doc]

**Additional file 1: Additional Methods**

## Minilab analysis of artemether/lumefantrine tablet products

Physical inspection was carried out on the tablets and packaging materials. Each tablet was examined for uniformity of colour, uniformity of shape, uniformity of size, dirty marks, presence of discolouration, presence of cracks, presence of strange smell, and presence of surface foreign matter. Packaging material of the tablets was also examined for the presence of spelling mistakes, manufacturing date, expiry date, manufacturer’s address, drug’s strength, number of unit dose, batch number and desired storage conditions.

For disintegration test, a 100 mL botttle was filled with tap (purified) water and heated on a hot plate until the temperature of the water reached 37 °C. A tablet was then placed in the warm water and stirred every 5 min for maximum of period of 30 min. This was repeated for six tablets for each batch of the drug product.

Thin-layer chromatography was performed for the assay of artemether and lumefantrine in the tablet products. A standard solution of artemether was prepared by crushing one tablet of the reference artemether tablet wrapped with aluminium foil using a pestle. The crushed reference standard in the aluminium foil was carefully emptied into a 25 mL glass bottle. All residual solids were washed down with 9 mL of methanol followed by 1 mL of glacial acetic acid. The glass bottle was closed and shaken for about 3 min, and the solution was allowed to stand for additional 5 min. The resulting solution (2 mg/mL) was used as the upper working limit standard. The lower working limit standard was prepared by transferring 4 mL of the upper working limit standard solution into a 10 mL vial and diluted with 1 mL of methanol. Similarly, a standard solution of lumefantrine was prepared by crushing one tablet of the reference lumefantrine tablet wrapped with aluminium foil using a pestle. The crushed reference standard in the aluminium foil was carefully emptied into a 100 mL glass bottle. All residual solids were washed down with 45 mL of methanol followed by 5 mL of glacial acetic acid. The glass bottle was closed and shaken for about 3 min, and the solution was allowed to stand for additional 5 min. 1 mL of the resulting standard stock solution was pipetted into a 10 mL vial and diluted with 2 mL of methanol, which was used as the upper working limit standard (0.8 mg/mL). The lower working limit standard solution (0.64 mg/mL) was prepared by pipetting 4 mL of the standard stock solution into a 25 mL vial and diluted with 11 mL of methanol.

Artemether and lumefantrine sample stock solutions were prepared by crushing one tablet containing ca. artemether/lumefantrine (20 mg/120 mg) wrapped in aluminium foil. The crushed artemether sample in the aluminium foil was carefully emptied into a 25 mL glass bottle. All residual solids were washed down with 5 mL of methanol followed by 0.55 mL of glacial acetic acid. The glass bottle was closed and shaken for about 3 min, and the solution was allowed to stand for additional 5 min. For artemether sample solution, 2.5 mL of the resulting stock solution was pipetted into a 10 mL vial and diluted with 2 mL of methanol. In the case of lumefantrine sample solution, 1 mL of the resulting stock sample solution was pipetted into a 40 mL vial and diluted with 23.5 mL of methanol followed by 2.5 mL of glacial acetic acid.

The upper working limit standard solution, lower working limit standard solution and the sample solution of artemether were spotted at different positions (1.5 cm from the bottom edge of the TLC) on the TLC using capillary tubes and allowed to dry. The mobile phase solution was prepared by mixing 18 mL of toluene, 4 mL of ethyl acetate and 2 mL of glacial acetic acid in a TLC chamber. The chamber was closed and mixed thoroughly. The chamber’s wall was lined with filter paper for 15 min. The TLC plate was then placed into the jar and closed. After 10 min the TLC plate was removed from the chamber and solvent front marked. The excess solvent was allowed to evaporate. To detect artemether, the TLC plate was immersed in sulphuric acid solution (10 mL of sulphuric acid dissolved in 190 mL of methanol), removed and dried. After drying, the visible artemether spots under daylight was marked with a pencil. The above procedure was repeated for lumefantrine with the following modifications: mobile phase solution was prepared by mixing 18 mL of ethyl acetate, 4 mL of methanol and 2 mL of glacial acetic acid in a TLC chamber. After drying, lumefantrine was detected at 254 nm using a UV lamp without immersion in any solution. Data analysis of the Minilab test was performed in accordance with the manufacturer’s instructions.

## Colorimetric analysis of artemether/lumefantrine tablet products

A standard artemether solution (24 mg/mL) was prepared by dissolving 120 mg of artemether in 5 mL glacial acetic acid. The resulting solution was further diluted to obtain concentrations of 12 mg/mL, 6 mg/mL and 3 mg/mL. A standard lumefantrine solution (14.4 mg/mL) was prepared by dissolving 144 mg of lumefantrine in 10 mL 10 % acetic acid in ethyl acetate. The resulting solution was further diluted to obtain concentrations of 7.2 mg/mL, 3.6 mg/mL and 1.8 mg/mL.

A mixed standard solution of artemether and lumefantrine was then prepared. First, 60 mg of artemether and 360 mg of lumefantrine standards were weighed and uniformly mixed on a white tile. A quantity of the mixed powder containing 48 mg of artemether was weighed and dissolved with glacial acetic acid to obtain a concentration of 24 mg/mL for artemether. The resulting solution was then diluted to obtain concentrations of 12 mg/mL, 6 mg/mL and 3 mg/mL for artemether. Similarly, a quantity of the mixed powder containing 60 mg of lumefantrine was weighed and dissolved with 10 % acetic acid in ethyl acetate obtain a concentration of 14.4 mg/mL for lumefantrine. The resulting solution was then diluted to obtain concentration of 7.2 mg/mL, 3.6 mg/mL and 1.8 mg/mL for lumefantrine.

Six (6) tablets of each artemether/lumefantrine tablet product were crushed into a fine powder. An equivalent amount of each powder containing 10 mg of artemether was weighed and shaken vigorously with 1 mL glacial acetic acid for 15 s and allowed to settle for 30 min. Similarly, an equivalent amount of each powder containing 60 mg of lumefantrine was weighed and shaken vigorously with 1 mL glacial acetic acid for 15 s and allowed to settle for 30 min. The resulting solution was diluted 1:10 with ethyl acetate. The sample solutions were prepared in triplicates. For the assay of artemether, 0.1 mL each of artemether standard, mixed artemether standard and artemether sample solutions were pipetted into a 24-well plate. 0.5 mL 85 % phosphoric acid was added to each well and mixed thoroughly. The 24-well plate was covered and placed in an oven at 35 ºC. The resulting colour was analysed with red versus blue pixel on the MVHimage colour software (MVHimagePCv8, Global Systems Science, University of California). For the assay of lumefantrine, 0.02 mL each of lumefantrine standard, mixed lumefantrine standard and lumefantrine sample solutions were pipetted into a 24-well plate. 0.5 mL methanol was added and mixed thoroughly. 0.25 mL of 1.1 M acetic acid was then added and mixed thoroughly. Next, 0.075 mL of Congo red was also added and thoroughly mixed. The plate was then covered and kept at 25 ºC for 20 min. The resulting colour was analysed with Red-Green-Blue (red %) pixel on the MVHimage colour software (MVHimagePCv8, Global Systems Science, University of California) (Fig. S1).

## CoDI analysis of artemether/lumefantrine tablet products

Specifically, each artemether/lumefantrine tablet was placed in the sample compartment from which a 405 nm laser beam is emitted. The intensity of light emitted through the tablet was recorded using a photoresistor coupled to a voltmeter. A blue filter was then placed over the photoresistor and the intensity rerecorded from the same tablet (Fig. S2). A CoDI value was calculated from the ratio of light intensity with and without the blue filter.

## HPLC analysis of artemether/lumefantrine tablet products

Artemether and lumefantrine reference standard solution was prepared by weighing 20 mg of artemether and 120 mg of lumefantrine reference substance in a beaker and dissolved with 10 mL of diluent (1 mL of TFA/500 mL of 20 mmol 1-hexanesulfonic acid sodium salt/500 mL of distilled water/1000 mL of ACN). This solution was then transferred into a 25 mL volumetric flask and diluted to the mark with diluent. The solution was sonicated in a warm water bath for 30 min. A portion of the reference standard solution was transferred into HPLC vials for analysis. The same procedure was used for the preparation of sample solutions by weighing an equivalent amount of each sample containing 20 mg of artemether and 120 mg of lumefantrine. A portion of the solution was then filtered through a PVDF syringe filter (0.45 µm, 30 mm, Millipore, USA) into HPLC vials for analysis.

The reference standard and sample solutions were analysed using HPLC chromatograph (Agilent Technologies 1260 infinity, Germany) equipped with online degasser, autosampler, pumping system, column compartment and UV detector. 20 μL of each solution was loaded via the autosampler onto a C_18_ reverse phase column (Zorbax Eclipse plus C_18_, 3.5 μm, 100 mm × 4.6 mm, Serial number: USAUXR07425, Princeton Chromatography Inc., USA). The following chromatographic conditions were used: flow rate; 1.0 mL/min, column temperature; 30 ˚C, detection wavelength; 210 nm and 380 nm for artemether and lumefantrine, respectively. Artemether and lumefantrine were eluted by a 25 min gradient (Table S1) using mobile phase comprising of solvent A (20 mmol 1-hexanesulfonic acid sodium salt (3.77 g into 1000 mL volumetric flask)/ACN/TFA (49:51:0.1 v/v/v)) and solvent B (ACN/TFA (100:0.1 v/v)).
