# Supplementary figures and images for: Usefulness of combined screening methods for rapid detection of falsified and/or substandard medicines in the absence of a confirmatory method
Source: Malar J. 2019 Dec 5;18:403. doi: 10.1186/s12936-019-3045-y (PMC6896689; doi:10.1186/s12936-019-3045-y)

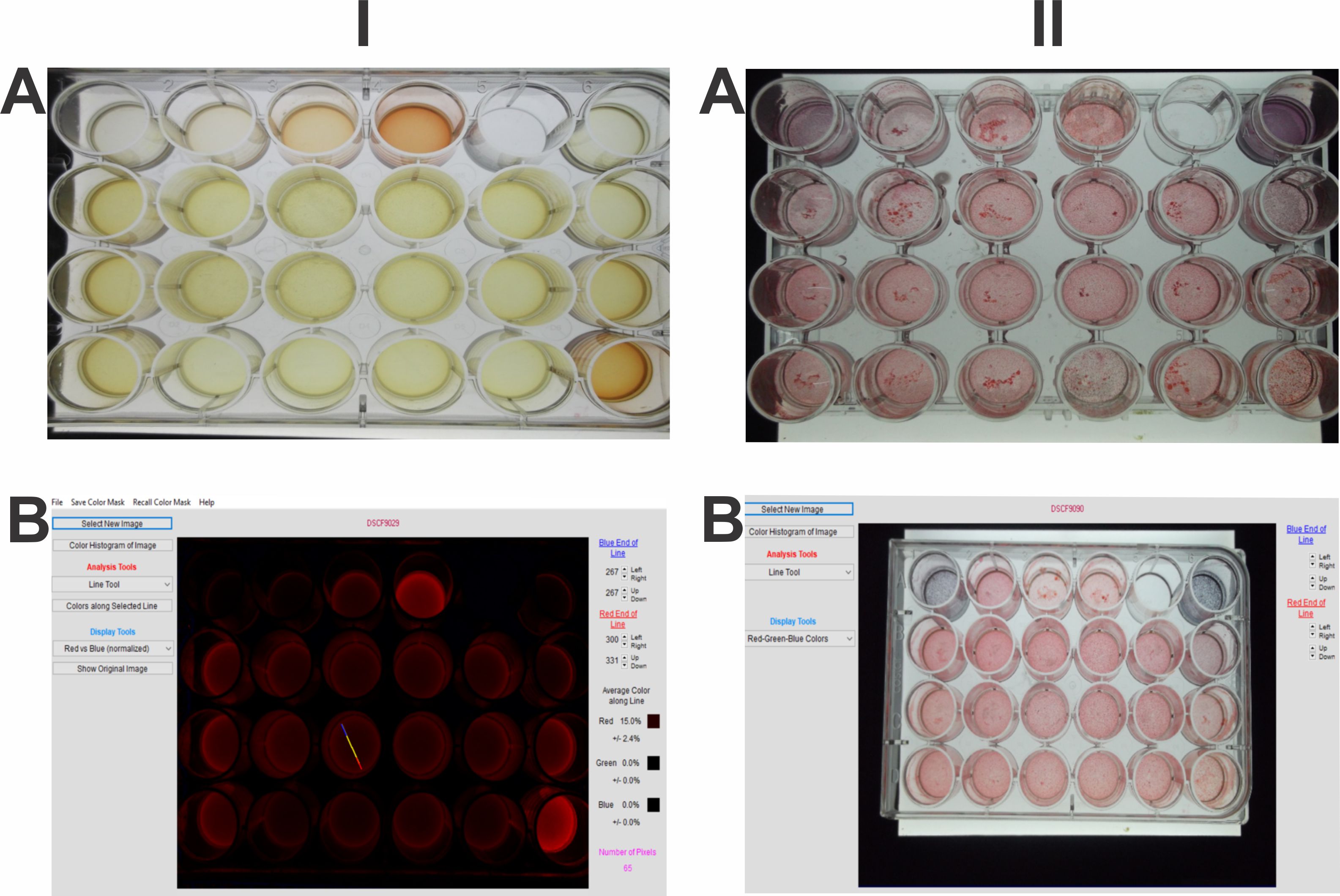

Supplement: Supplementary file 2 — Additional file 2: Figure S1. Colorimetric analysis of artemether/lumefantrine tablet products. IA: Samples in the 24-well plate before analysis with the colour software for artemether. IIA: Samples in the 24-well plate before analysis with the colour software for lumefantrine. IB: Samples in the 24-well plate during analysis with the MVHImage software for artemether. IIB: Samples in the 24-well plate during the analysis with the MVHImage software for lumefantrine. [file 12936_2019_3045_MOESM2_ESM.jpg]

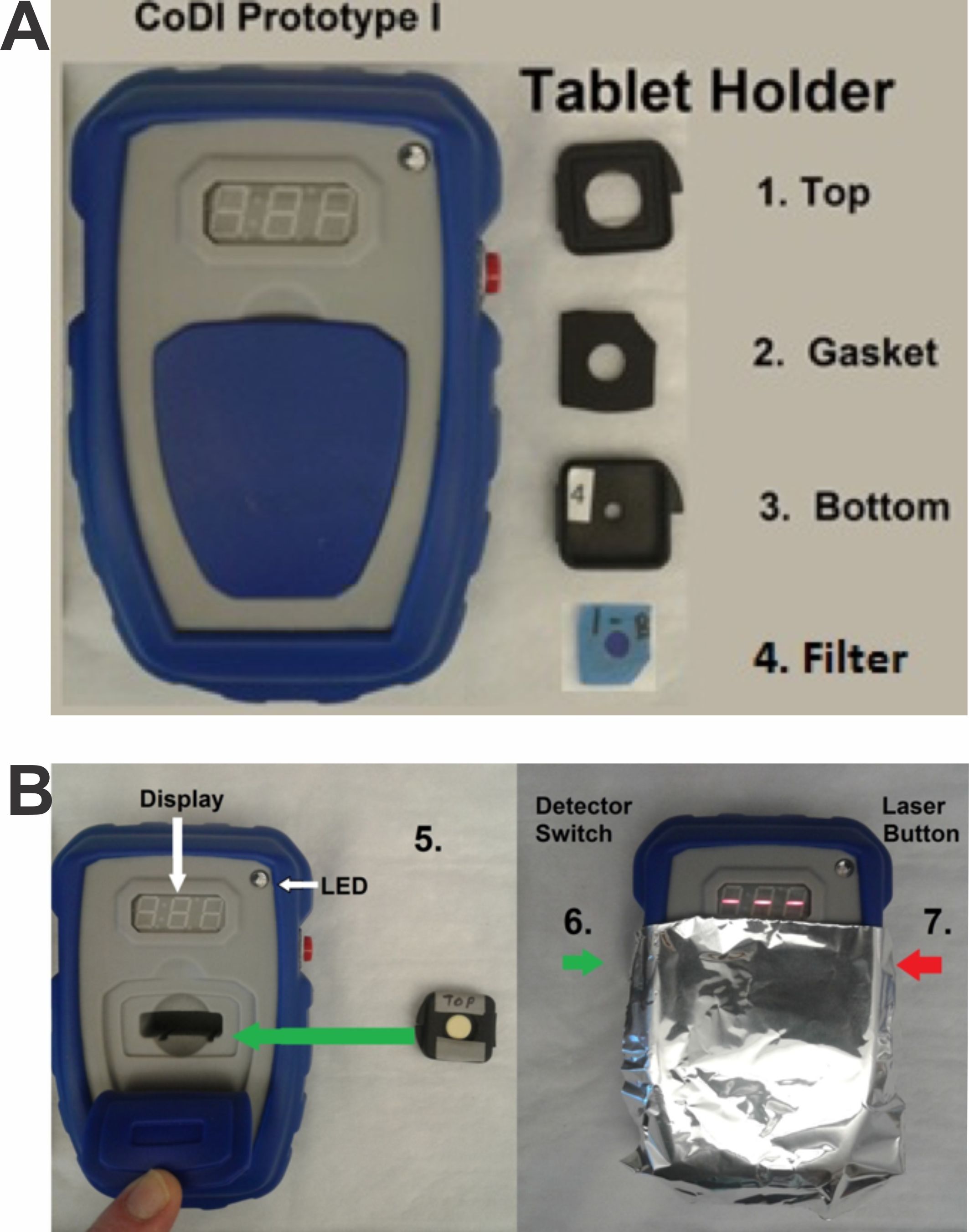

Supplement: Supplementary file 3 — Additional file 3: Figure S2. The prototype CoDI equipment. A: Different parts of the CoDI equipment. B: The CoDI equipment showing the slot and its operation. [file 12936_2019_3045_MOESM3_ESM.jpg]
