# Supplementary material for: Usefulness of combined screening methods for rapid detection of falsified and/or substandard medicines in the absence of a confirmatory method
Source: Malar J. 2019 Dec 5;18:403. doi: 10.1186/s12936-019-3045-y (PMC6896689; doi:10.1186/s12936-019-3045-y)
Supplement: Supplementary file 4 — Additional file 4: Table S1. Gradient elution programme. [file 12936_2019_3045_MOESM4_ESM.doc]

**Additional file 4: Table S1.** Gradient elution programme

| **Time (min)** | **Solvent A (%)** | **Solvent B (%)** |
| --- | --- | --- |
| 0.0 | 100 | 0 |
| 12.0 | 100 | 0 |
| 16.0 | 48 | 52 |
| 20.0 | 48 | 52 |
| 20.1 | 100 | 0 |
| 25.0 | 100 | 0 |
