# Supplementary material for: Usefulness of combined screening methods for rapid detection of falsified and/or substandard medicines in the absence of a confirmatory method
Source: Malar J. 2019 Dec 5;18:403. doi: 10.1186/s12936-019-3045-y (PMC6896689; doi:10.1186/s12936-019-3045-y)
Supplement: Supplementary file 5 — Additional file 5: Table S2. Minilab test results for artemether/lumefantrine tablets of unknown quality [file 12936_2019_3045_MOESM5_ESM.docx]

**Additional file 5: Table S2.** Minilab test results for artemether/lumefantrine tablets of unknown quality

| **Drug Code** | **Batch** | **Active Ingredient** | **Tablet Defect** | **Packing Defect** | **Av. Disintegration Time**  **NMT 30 min** | **Rf^a)^** | **Rf^b)^** | **Error^c)^ NMT 5 %** | **Shape^d)^** | **Intensity^e)^** | **Conclusion** |
| --- | --- | --- | --- | --- | --- | --- | --- | --- | --- | --- | --- |
| AT | 1 | Artemether | No defect | No defect | 5.0 | 0.657 | 0.644 | 2.0 | + | + | Passed |
|  |  | Lumefantrine |  |  |  | 0.841 | 0.841 | 0.0 | + | + | Passed |
|  | 2 | Artemether | No defect | No defect | 5.0 | 0.702 | 0.696 | 0.9 | + | + | Passed |
|  |  | Lumefantrine |  |  |  | 0.857 | 0.857 | 0.0 | + | + | Passed |
|  | 3 | Artemether | No defect | No defect | 5.0 | 0.696 | 0.711 | 2.2 | + | + | Passed |
|  |  | Lumefantrine |  |  |  | 0.831 | 0.831 | 0.0 | + | + | Passed |
| CG | 1 | Artemether | No defect | No defect | 5.0 | 0.659 | 0.659 | 0.0 | + | + | Passed |
|  |  | Lumefantrine |  |  |  | 0.905 | 0.924 | 2.1 | + | + | Passed |
|  | 2 | Artemether | No defect | No defect | 5.0 | 0.671 | 0.671 | 0.0 | + | + | Passed |
|  |  | Lumefantrine |  |  |  | 0.864 | 0.864 | 0.0 | + | + | Passed |
|  | 3 | Artemether | No defect | No defect | 5.0 | 0.700 | 0.700 | 0.0 | + | + | Passed |
|  |  | Lumefantrine |  |  |  | 0.866 | 0.866 | 0.0 | + | + | Passed |
| CD | 1 | Artemether | No defect | No defect | 5.0 | 0.719 | 0.705 | 2.0 | + | + | Passed |
|  |  | Lumefantrine |  |  |  | 0.895 | 0.895 | 0.0 | + | + | Passed |
|  | 2 | Artemether | No defect | No defect | 5.0 | 0.634 | 0.634 | 0.0 | + | + | Passed |
|  |  | Lumefantrine |  |  |  | 0.843 | 0.859 | 1.9 | + | + | Passed |
|  | 3 | Artemether | No defect | No defect | 5.0 | 0.890 | 0.890 | 0.0 | + | + | Passed |
|  |  | Lumefantrine |  |  |  | 0.890 | 0.890 | 0.0 | + | + | Passed |
| CO | 1 | Artemether | No defect | No defect | 1.0 | 0.653 | 0.667 | 2.1 | + | + | Passed |
|  |  | Lumefantrine |  |  |  | 0.871 | 0.879 | 0.9 | + | + | Passed |
|  | 2 | Artemether | No defect | No defect | 1.0 | 0.676 | 0.676 | 0.0 | + | + | Passed |
|  |  | Lumefantrine |  |  |  | 0.887 | 0.871 | 1.8 | + | + | Passed |
|  | 3 | Artemether | No defect | No defect | 1.0 | 0.729 | 0.743 | 1.4 | + | + | Passed |
|  |  | Lumefantrine |  |  |  | 0.873 | 0.873 | 0.0 | + | + | Passed |
| LO | 1 | Artemether | No defect | No defect | 5.0 | 0.671 | 0.671 | 0.0 | + | + | Passed |
|  |  | Lumefantrine |  |  |  | 0.871 | 0.879 | 0.9 | + | + | Passed |
|  | 2 | Artemether | No defect | No defect | 5.0 | 0.662 | 0.699 | 0.7 | + | + | Passed |
|  |  | Lumefantrine |  |  |  | 0.887 | 0.871 | 1.8 | + | + | Passed |
|  | 3 | Artemether | No defect | No defect | 5.0 | 0.685 | 0.685 | 0.0 | + | + | Passed |
|  |  | Lumefantrine |  |  |  | 0.873 | 0.873 | 0.0 | + | + | Passed |
| DA | 1 | Artemether | No defect | No defect | 5.0 | 0.618 | 0.611 | 1.1 | + | + | Passed |
|  |  | Lumefantrine |  |  |  | 0.78 | 0.78 | 0.0 | + | + | Passed |
|  | 2 | Artemether | No defect | No defect | 5.0 | 0.630 | 0.637 | 1.1 | + | + | Passed |
|  |  | Lumefantrine |  |  |  | 0.820 | 0.820 | 0.0 | + | + | Passed |
|  | 3 | Artemether | No defect | No defect | 5.0 | 0.614 | 0.614 | 0.0 | + | + | Passed |
|  |  | Lumefantrine |  |  |  | 0.803 | 0.803 | 0.0 | + | + | Passed |
| GM | 1 | Artemether | No defect | No defect | 5.0 | 0.601 | 0.607 | 1.0 | + | + | Passed |
|  |  | Lumefantrine |  |  |  | 0.794 | 0.794 | 0.0 | + | + | Passed |
|  | 2 | Artemether | No defect | No defect | 5.0 | 0.667 | 0.652 | 1.4 | + | + | Passed |
|  |  | Lumefantrine |  |  |  | 0.757 | 0.757 | 0.0 | + | + | Passed |
| CV | 1 | Artemether | No defect | No defect | 51.0 | 0.680 | nd | na | * | * | Failed |
|  |  | Lumefantrine |  |  |  | 0.884 | nd | na | * | * | Failed |
| CW | 1 | Artemether | No defect | No defect | 39.0 | 0.650 | nd | na | * | * | Failed |
|  |  | Lumefantrine |  |  |  | 0.921 | nd | na | * | * | Failed |
| CX | 1 | Artemether | No defect | No defect | 19.0 | 0.640 | 0.640 | 0 | + | + | Passed |
|  |  | Lumefantrine |  |  |  | 0.870 | 0.870 | 0 | + | + | Passed |
| CY | 1 | Artemether | Surface cavities | No defect | > 60.0 | 0.650 | nd | na | * | * | Failed |
|  |  | Lumefantrine |  |  |  | 0.824 | nd | na | * | * | Failed |
| CZ | 1 | Artemether | No defect | No defect | 8.0 | 0.720 | 0.720 | 0 | + | + | Passed |
|  |  | Lumefantrine |  |  |  | 0.838 | 0.838 | 0 | + | + | Passed |

^a)^Rf of the standard

^b)^Rf of the test sample

^c)^Sampling error

^d)^Shape of the test sample spot

^e)^Intensity of the test sample spot

^+^Shape and intensity of the test spot similar to that of the standard spot

NMT: Not more than

nd: not detected

na: not applicable

*Absence of shape and intensity of the test spot
