# Supplementary material for: Usefulness of combined screening methods for rapid detection of falsified and/or substandard medicines in the absence of a confirmatory method
Source: Malar J. 2019 Dec 5;18:403. doi: 10.1186/s12936-019-3045-y (PMC6896689; doi:10.1186/s12936-019-3045-y)
Supplement: Supplementary file 6 — Additional file 6: Table S3. Assay of artemether/lumefantrine tablets of unknown quality using colorimetry. [file 12936_2019_3045_MOESM6_ESM.docx]

## Additional file 6: Table S3 Assay of artemether/lumefantrine tablets of unknown quality using colorimetry

| **Drug Code** | **Batch** | **Active Ingredient** | **Assay content (%)** | **Conclusion**  **90.00-110.00 %** |
| --- | --- | --- | --- | --- |
| AT | 1 | Artemether | 93.84 | Passed |
|  |  | Lumefantrine | 97.36 | Passed |
|  | 2 | Artemether | 94.60 | Passed |
|  |  | Lumefantrine | 91.70 | Passed |
|  | 3 | Artemether | 90.70 | Passed |
|  |  | Lumefantrine | 90.20 | Passed |
| CG | 1 | Artemether | 99.80 | Passed |
|  |  | Lumefantrine | 90.40 | Passed |
|  | 2 | Artemether | 90.17 | Passed |
|  |  | Lumefantrine | 86.70 | Failed |
|  | 3 | Artemether | 101.03 | Passed |
|  |  | Lumefantrine | 98.00 | Passed |
| CD | 1 | Artemether | 92.40 | Passed |
|  |  | Lumefantrine | 103.93 | Passed |
|  | 2 | Artemether | 104.20 | Passed |
|  |  | Lumefantrine | 101.86 | Passed |
|  | 3 | Artemether | 95.40 | Passed |
|  |  | Lumefantrine | 102.52 | Passed |
| CO | 1 | Artemether | 91.72 | Passed |
|  |  | Lumefantrine | 90.36 | Passed |
|  | 2 | Artemether | 97.60 | Passed |
|  |  | Lumefantrine | 92.71 | Passed |
|  | 3 | Artemether | 93.60 | Passed |
|  |  | Lumefantrine | 96.03 | Passed |
| LO | 1 | Artemether | 99.50 | Passed |
|  |  | Lumefantrine | 93.05 | Passed |
|  | 2 | Artemether | 100.60 | Passed |
|  |  | Lumefantrine | 91.10 | Passed |
|  | 3 | Artemether | 101.62 | Passed |
|  |  | Lumefantrine | 90.23 | Passed |
| DA | 1 | Artemether | 105.67 | Passed |
|  |  | Lumefantrine | 96.20 | Passed |
|  | 2 | Artemether | 101.51 | Passed |
|  |  | Lumefantrine | 92.23 | Passed |
|  | 3 | Artemether | 100.85 | Passed |
|  |  | Lumefantrine | 91.10 | Passed |
| GM | 1 | Artemether | 86.00 | Failed |
|  |  | Lumefantrine | 84.73 | Failed |
|  | 2 | Artemether | 91.70 | Passed |
|  |  | Lumefantrine | 93.70 | Passed |
