# Supplementary material for: Usefulness of combined screening methods for rapid detection of falsified and/or substandard medicines in the absence of a confirmatory method
Source: Malar J. 2019 Dec 5;18:403. doi: 10.1186/s12936-019-3045-y (PMC6896689; doi:10.1186/s12936-019-3045-y)
Supplement: Supplementary file 7 — Additional file 7: Table S4. Assay of artemether/lumefantrine tablets of unknown quality using Counterfeit Detection Indicator (CoDI). [file 12936_2019_3045_MOESM7_ESM.docx]

**Additional file 7: Table S4** Assay of artemether/lumefantrine tablets of unknown quality using Counterfeit Detection Indicator (CoDI)

| **Drug Code** | **Batch** | **Active Ingredient** | **CoDI value** | **Conclusion**  **0.44-0.68** |
| --- | --- | --- | --- | --- |
| AT | 1 | Artemether | na | - |
|  |  | Lumefantrine |  |  |
|  | 2 | Artemether | na | - |
|  |  | Lumefantrine |  |  |
|  | 3 | Artemether | na | - |
|  |  | Lumefantrine |  |  |
| CG | 1 | Artemether | 0.59 | Passed |
|  |  | Lumefantrine |  |  |
|  | 2 | Artemether | 0.60 | Passed |
|  |  | Lumefantrine |  |  |
|  | 3 | Artemether | 0.60 | Passed |
|  |  | Lumefantrine |  |  |
| CD | 1 | Artemether | na | - |
|  |  | Lumefantrine |  |  |
|  | 2 | Artemether | na | - |
|  |  | Lumefantrine |  |  |
|  | 3 | Artemether | na | - |
|  |  | Lumefantrine |  |  |
| CO | 1 | Artemether | na | - |
|  |  | Lumefantrine |  |  |
|  | 2 | Artemether | na | - |
|  |  | Lumefantrine |  |  |
|  | 3 | Artemether | na | - |
|  |  | Lumefantrine |  |  |
| LO | 1 | Artemether | na | - |
|  |  | Lumefantrine |  |  |
|  | 2 | Artemether | na | - |
|  |  | Lumefantrine |  |  |
|  | 3 | Artemether | na | - |
|  |  | Lumefantrine |  |  |
| DA | 1 | Artemether | na | - |
|  |  | Lumefantrine |  |  |
|  | 2 | Artemether | na | - |
|  |  | Lumefantrine |  |  |
|  | 3 | Artemether | na | - |
|  |  | Lumefantrine |  |  |
| GM | 1 | Artemether | na | - |
|  |  | Lumefantrine |  |  |
|  | 2 | Artemether | na | - |
|  |  | Lumefantrine |  |  |

na: Not applicable
