# Supplementary material for: Usefulness of combined screening methods for rapid detection of falsified and/or substandard medicines in the absence of a confirmatory method
Source: Malar J. 2019 Dec 5;18:403. doi: 10.1186/s12936-019-3045-y (PMC6896689; doi:10.1186/s12936-019-3045-y)
Supplement: Supplementary file 8 — Additional file 8: Table S5. Assay of artemether/lumefantrine tablets of unknown quality using HPLC. [file 12936_2019_3045_MOESM8_ESM.docx]

## Additional file 8: Table S5 Assay of artemether/lumefantrine tablets of unknown quality using HPLC

| **Drug Code** | **Batch** | **Active Ingredient** | **Assay content (%) ± RSD** | **Conclusion**  **90.00-110.00 %** |
| --- | --- | --- | --- | --- |
| AT | 1 | Artemether | 97.95 ± 2.29 | Passed |
|  |  | Lumefantrine | 104.73 ± 0.03 | Passed |
|  | 2 | Artemether | 92.60 ± 3.83 | Passed |
|  |  | Lumefantrine | 94.64 ± 0.35 | Passed |
|  | 3 | Artemether | 97.31 ± 3.75 | Passed |
|  |  | Lumefantrine | 97.80 ± 0.05 | Passed |
| CG | 1 | Artemether | 101.38 ± 4.15 | Passed |
|  |  | Lumefantrine | 95.32 ± 0.97 | Passed |
|  | 2 | Artemether | 83.85 ± 1.23 | Failed |
|  |  | Lumefantrine | 83.06 ± 0.09 | Failed |
|  | 3 | Artemether | 90.62 ± 1.53 | Passed |
|  |  | Lumefantrine | 91.28 ± 0.03 | Passed |
| CD | 1 | Artemether | 93.56 ± 1.18 | Passed |
|  |  | Lumefantrine | 97.82 ± 0.25 | Passed |
|  | 2 | Artemether | 93.14 ± 1.04 | Passed |
|  |  | Lumefantrine | 98.03 ± 0.15 | Passed |
|  | 3 | Artemether | 95.32 ± 3.53 | Passed |
|  |  | Lumefantrine | 98.39 ± 0.06 | Passed |
| CO | 1 | Artemether | 95.12 ± 1.45 | Passed |
|  |  | Lumefantrine | 95.01 ± 0.04 | Passed |
|  | 2 | Artemether | 99.44 ± 0.20 | Passed |
|  |  | Lumefantrine | 93.16 ± 0.12 | Passed |
|  | 3 | Artemether | 106.92 ± 0.74 | Passed |
|  |  | Lumefantrine | 96.78 ± 0.06 | Passed |
| LO | 1 | Artemether | 95.77 ± 1.88 | Passed |
|  |  | Lumefantrine | 100.71 ± 0.84 | Passed |
|  | 2 | Artemether | 95.54 ± 1.82 | Passed |
|  |  | Lumefantrine | 101.46 ± 0.11 | Passed |
|  | 3 | Artemether | 119.38 ± 1.25 | Failed |
|  |  | Lumefantrine | 106.31 ± 0.12 | Passed |
| DA | 1 | Artemether | 100.11 ± 4.51 | Passed |
|  |  | Lumefantrine | 93.36 ± 0.07 | Passed |
|  | 2 | Artemether | 98.62 ± 1.09 | Passed |
|  |  | Lumefantrine | 90.45 ± 0.69 | Passed |
|  | 3 | Artemether | 95.13 ± 1.66 | Passed |
|  |  | Lumefantrine | 91.01 ± 4.6 | Passed |
| GM | 1 | Artemether | 89.25 ± 2.29 | Failed |
|  |  | Lumefantrine | 82.17 ± 2.06 | Failed |
|  | 2 | Artemether | 102.19 ± 0.32 | Passed |
|  |  | Lumefantrine | 90.96 ± 0.10 | Passed |

RSD: Relative standard deviation
